# Characteristics and outcomes of primary and secondary resistance to immune checkpoint inhibitors in hepatocellular carcinoma

**DOI:** 10.1007/s00262-025-04089-x

**Published:** 2025-06-07

**Authors:** Xiaowen Cui, Minghao Ruan, Yao Li, Cheng Yang, Jin Zhang, Riming Jin, Dong Wu, Wen Sun, Ruoyu Wang

**Affiliations:** 1 Department of Oncology, Eastern Hepatobiliary Surgery Hospital, The Naval Medical University, Shanghai, China; 2 The First Department of Hepatic Surgery, Eastern Hepatobiliary Surgery Hospital, The Naval Medical University, 225 Changhai Road, Shanghai, 200438 China; 3 Department of Special Treatment I and Liver Transplantation, Eastern Hepatobiliary Surgery Hospital, The Naval Medical University, Shanghai, China; 4 National Center for Liver Cancer, The Naval Medical University, 800 Xiangyin Road, Shanghai, 200433 China

**Keywords:** Primary resistance, Secondary resistance, Immune checkpoint inhibitors, Hepatocellular carcinoma, Post-progression survival

## Abstract

Resistance limits the efficacy and durability of immune checkpoint inhibitors (ICIs) in hepatocellular carcinoma (HCC). Therefore, we conducted a retrospective cohort study to investigate the outcomes and characteristics of HCC patients with resistance to immunotherapy. Patients with HCC who have received ICIs at Eastern Hepatobiliary Surgery Hospital between 2016 and 2021 were retrospectively screened and divided into primary resistance, secondary resistance, and durable response group. Time to progression (TTP), overall survival (OS), subsequent management and post-progression survival (PPS) were analyzed. Of 496 patients included, 229 (46.2%) and 141 (28.4%) patients developed primary and secondary resistance, and 126 (25.4%) patients achieved a durable response, the median TTP was 2.83 [2.56–3.09] months, 11.93 [10.45–13.40] months, and not reached, respectively, whereas the median OS was 12.83 [10.36–15.30] months, 31.53 [28.09–34.97] and not reached, respectively. Multivariate logistic regression revealed that Child–Pugh score, BCLC stage, and combined systemic therapies (ICI plus bevacizumab or lenvatinib versus ICI monotherapy) were independently associated with primary resistance, and only combined systemic therapies (ICI plus bevacizumab versus ICI monotherapy) were independently associated with secondary resistance. AFP levels were independently associated with PPS in patients with primary resistance, while post-progression therapies (ICI-based therapies versus others) were independently associated with PPS in patients with resistance. The risk of resistance was notably lower in patients receiving the combination of ICI plus bevacizumab. High AFP levels were associated with the survival of patients with primary resistance. ICI-based maintenance therapy after resistance may provide a significant survival advantage for HCC patients.

**Supplementary Information:**

The online version contains supplementary material available at 10.1007/s00262-025-04089-x.

## Introduction

The first-line therapy for advanced hepatocellular carcinoma (HCC) used to be the tyrosine kinase inhibitor (TKI) sorafenib, which has remained unchanged for almost a decade since its approval in 2007 [1]. In 2018, lenvatinib was approved as a non-inferior option to sorafenib [2]. With the advent of immune checkpoint inhibitors (ICIs), the therapeutic landscape for HCC is rapidly evolving [3]. The IMbrave 150 trial demonstrated that the regimen of atezolizumab plus bevacizumab displayed superior clinical outcomes to sorafenib [4, 5]. Additionally, the HIMALAYA phase 3 trial provides tremelimumab and durvalumab as an alternative regimen for advanced HCC [6]. On the other hand, another anti-VEGF-based approach via multi-target TKIs also synergistically enhances the response to ICI [7, 8]. The combination of lenvatinib and pembrolizumab achieved a promising antitumor activity in the phase 1b study [7]. Although the same success was neither seen in the later LEAP-002 [9] nor COMIC-132 trial [8], the combination of ICI plus TKI still has an important role in HCC.

Moreover, resistance remains a significant issue for HCC patients despite impressive advances in immunotherapy-based treatments. Meanwhile, resistance can be categorized as primary resistance (ineffective to immunotherapy) and secondary resistance (progression after an initial response) [10], with distinct outcomes and molecular mechanisms [11]. Multiple mechanisms and determinants influence immunotherapy resistance of HCC, including intrinsic and extrinsic factors [12]. Although several studies have investigated the mechanism of immunotherapy resistance in HCC, they are mostly based on cellular experiments and animal models, lacking detailed clinical characteristics of patients with resistance from the real world [11, 13–15]. Currently, early identification of resistance risks before ICI treatment and sequential therapies to manage immunotherapy resistance are still unsolved issues for HCC. Therefore, we conducted a retrospective cohort study to investigate the outcomes and characteristics of patients with primary or secondary resistance to immunotherapy in HCC. We hypothesized that, compared to monotherapy, ICI combination therapies (e.g., with bevacizumab or lenvatinib) would lower the risk of both primary and secondary resistance and that clinicopathological factors would predict resistance outcomes.

## Patients and methods

### Study design and patients

This is a single-center retrospective cohort study conducted at Eastern Hepatobiliary Surgery Hospital (EHBH). HCC patients who received ICI therapy (anti-PD-1/PD-L1 mAb) at EHBH between 2016 and 2021 were enrolled. Patients were recruited from September 2016 to December 2021, with ICI exposure starting at treatment initiation, follow-up continuing until death or data cutoff (October 2022), and data collected from medical records between July and October 2022. Inclusion criteria were as follows: (1) patients aged 18–75 years; (2) pathologically or clinically diagnosed HCC; (3) have received at least six weeks of exposure to ICI therapy; (4) with an Eastern Cooperative Oncology Group (ECOG) performance score 0–1, and a Child–Pugh score A or B; (5) had at least one measurable lesion and tumor response was assessed by Response Evaluation Criteria in Solid Tumors (RECIST) version 1.1. Exclusion criteria included: (1) response unevaluable; (2) first response evaluation > six months from ICI initiation; (3) follow-up < six months without PD; (4) discontinuation of ICI treatment without PD.

According to the definition of tumor resistance to PD-1 pathway blockade recommended by the Society for Immunotherapy of Cancer (SITC) Immunotherapy Resistance Taskforce [10], patients were divided into primary resistance, secondary resistance, and durable response group. Briefly, primary resistance was defined as when HCC patients were exposed to ICI therapy for at least six weeks and with the best overall response (BOR) of PD or SD for less than six months. Secondary resistance was defined as HCC patients having disease progression after the initial objective response (CR/PR) or SD for more than six months. The durable response group consists of patients with the durable response (CR, PR, or SD) for more than six months without progression at data cutoff. The baseline characteristics of patients were extracted from medical records. The study was conducted following the Declaration of Helsinki (as revised in 2013) and approved by the Ethics Committee of EHBH. Due to the retrospective nature of this study, informed consent was waived by the Ethics Committee. Patients WERE NOT involved in the design, or conduct, or reporting, or dissemination plans of our research.

### Objectives and assessments

The primary objective was to compare the outcome (time to progression (TTP) and overall survival (OS)) of HCC patients with primary resistance, secondary resistance, or durable response to ICI therapy, as well as the clinicopathological factors associated with resistance. TTP was defined as the time interval between ICI initiation and tumor progression. OS was defined as the time interval between ICI initiation and death from any cause. Clinicopathological factors were compared between patients with primary resistance and secondary resistance/durable response or between patients with secondary resistance and durable response. Subsequent management post-resistance was also obtained and was classified as ICI-based therapy and other therapy (including locoregional therapy (LRT), TKIs, or best supportive care (BSC)). Post-progression survival (PPS) was compared in patients with a follow-up ≥ one-month post-resistance. PPS was defined as the time interval between the first documentary tumor progression after immunotherapy to death from any cause. Confounders included HBV status, prior therapies, and ECOG PS; effect modifiers included age and AFP levels. HCC was diagnosed per AASLD guidelines [16] (pathological or imaging-based). AFP and DCP were dichotomized at 400 μg/L based on prior literature [17–20]. The data were collected and analyzed from July to October 2022.

### Statistical analyses

Categorical variables were expressed as numbers (percentages) and compared with odds ratio (OR) using univariate logistic regression analysis and multivariate logistic regression analysis. TTP, OS, and PPS were analyzed by the Kaplan–Meier method and compared by the Log-rank method. The median TTP, OS, or PPS was expressed as numbers [95% CI]. The PPS was further analyzed by the COX proportional hazards model. Time-adjusted Cox regression analysis was conducted to assess the effect of resistance on OS, and *p* values were calculated from the Wald test. *p* < 0.05 was considered statistically significant. Stata 16 for Windows software was used for statistical analysis.

## Results

### Patients and outcomes

**Fig. 1:**
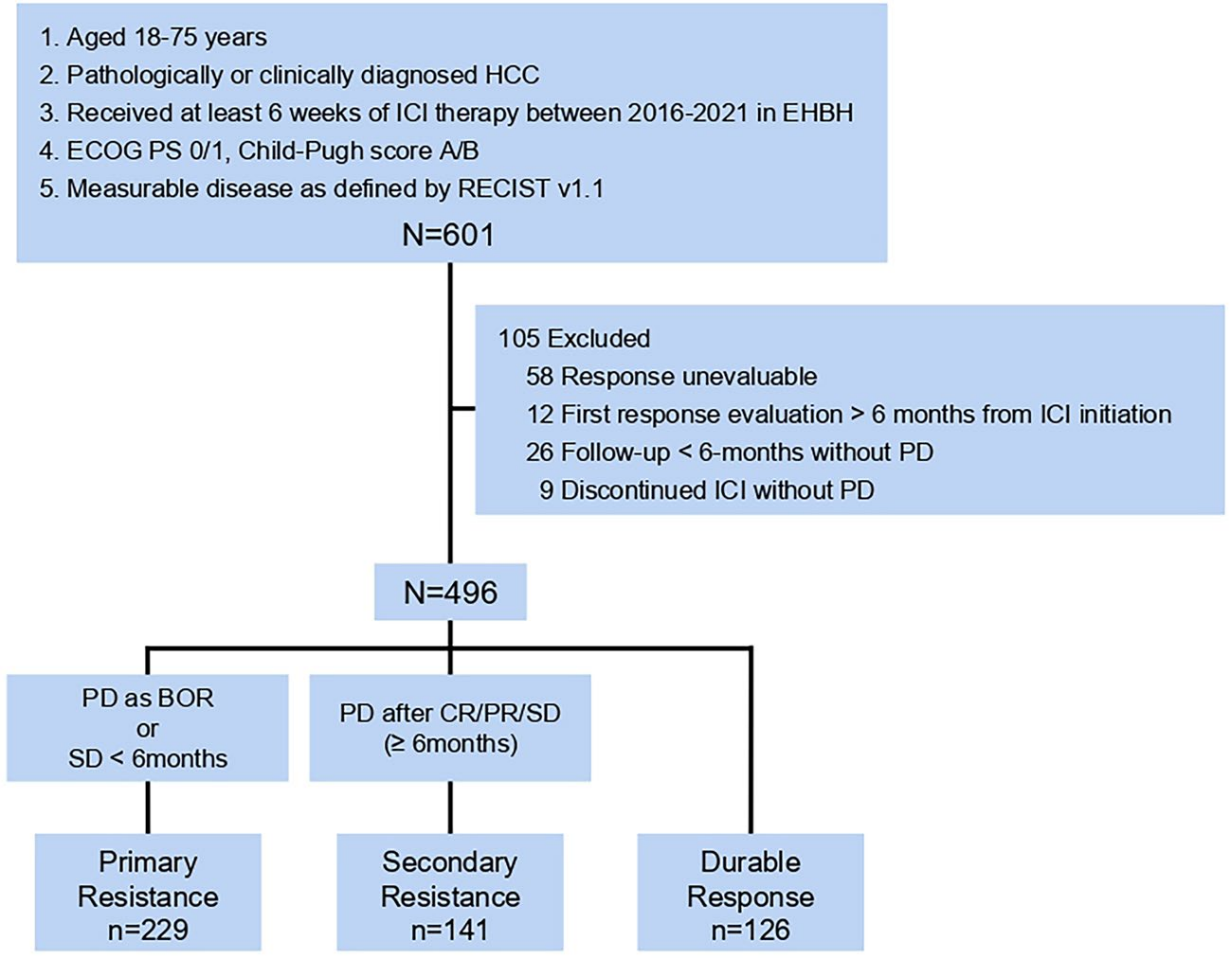
Study flow chart

This study enrolled 601 patients with HCC who met the inclusion criteria (Fig. 1). One-hundred and five patients were excluded (58 with response unevaluable; 12 with first response evaluation > six months from ICI initiation; 26 with follow-up < six months without PD; 9 with discontinuation of ICI treatment without PD). Of the remaining 496 patients in the cohort, 229 patients (46.2%) developed primary resistance, 141 patients (28.4%) developed secondary resistance, and 126 patients (25.4%) achieved durable responses (Fig. 1). The demographic and baseline characteristics of patients are shown in Table 1. 169 (73.7%), 110 (78.0%), and 111 patients (88.0%) have received the combination therapy of LRT (local regional treatment) and ICIs in primary resistance, secondary resistance, and durable response groups, respectively. In addition, in primary resistance, secondary resistance, and durable response groups, 173 (75.6%), 119 (84.4%) and 113 patients (89.7%) were treated with the combination of systemic therapies (bevacizumab, lenvatinib, etc.) and ICIs, respectively.

**Table 1:** Baseline characteristics of patients

Characteristic	Primary resistance (n = 229)	Secondary resistance (n = 141)	Durable response (n = 126)	*p* value
*Age, years*				0.07
≥ 60	48 (20.9%)	42 (29.7%)	24 (19.0%)	
< 60	181 (79.0%)	99 (70.2%)	102 (80.9%)	
*Gender*				0.806
Male	201 (87.7%)	121 (85.8%)	108 (85.7%)	
Female	28 (12.2%)	20 (14.1%)	18 (14.2%)	
*HBV*				0.374
Yes	220 (96.0%)	132 (93.6%)	117 (92.8%)	
No	9 (3.9%)	9 (6.3%)	9 (7.1%)	
*HBsAg*				0.028
Yes	216 (94.3%)	123 (87.2%)	110 (87.3%)	
No	13 (5.6%)	18 (12.7%)	16 (12.6%)	
*HCV*				0.142
Yes	3 (1.3%)	5 (3.5%)	6 (4.76%)	
No	226 (98.6%)	136 (96.4%)	120 (95.2%)	
*ECOG PS*				0.159
0	152 (66.3%)	93 (65.9%)	95 (75.3%)	
1	77 (33.6%)	48 (34.0%)	31 (24.6%)	
*Child–pugh score*				0.002
A	200 (87.3%)	135 (95.7%)	121 (96.0%)	
B	29 (12.6%)	6 (4.2%)	5 (3.9%)	
*Macrovascular invasion*				0.188
Yes	108 (47.1%)	53 (37.5%)	53 (42.0%)	
No	121 (52.8%)	88 (62.4%)	73 (57.9%)	
*Extrahepatic spread*				0.099
Yes	76 (33.1%)	47 (33.3%)	29 (23.0%)	
No	153 (66.8%)	94 (66.6%)	97 (76.9%)	
*BCLC stage*				0.001
A	15 (6.5%)	20 (14.1%)	27 (21.4%)	
B	65 (28.3%)	39 (27.6%)	25 (19.8%)	
C	149 (65.0%)	82 (58.1%)	74 (58.7%)	
*AFP, μg/L*				0.007
≥ 400	112 (48.9%)	46 (32.6%)	57 (45.2%)	
< 400	117 (51.0%)	95 (67.3%)	65 (51.5%)	
*DCP, mAU/mL*				0.011
≥ 400	156 (68.1%)	74 (52.4%)	74 (58.7%)	
< 400	72 (31.4%)	66 (46.8%)	46 (36.5%)	
*First line*				0.559
Yes	190 (82.9%)	119 (84.3%)	110 (87.3%)	
No	39 (17.0%)	22 (15.6%)	16 (12.6%)	
*Treatment naive*				< 0.001
Yes	96 (41.9%)	59 (41.8%)	81 (64.2%)	
No	133 (58.0%)	82 (58.1%)	45 (35.7%)	
*LRT*				0.007
Yes	169 (73.7%)	110 (78.0%)	111 (88.0%)	
No	60 (26.2%)	31 (21.9%)	15 (11.9%)	
*Systematic therapies*				0.003
Yes	173(75.6%)	119(84.4%)	113(89.7%)	
Bevacizumab	4 (1.7%)	2 (1.4%)	16 (12.6%)	
Lenvatinib	120 (52.4%)	98 (69.5%)	86 (68.2%)	
Sorafenib	39 (17.0%)	12 (8.5%)	6 (4.7%)	
Other	10 (4.3%)	7 (4.9%)	5 (3.9%)	
No	56 (24.4%)	22 (15.6%)	13 (10.3%)	
*Best overall response*				< 0.001
CR	0	6 (4.2%)	2 (1.5%)	
PR	7 (3.0%)	34 (24.1%)	58 (46.0%)	
SD	35 (15.2%)	101 (71.6%)	66 (52.3%)	
PD	187 (81.6%)	0	0	
Previous therapies				0.922
Pre-Surgery	88 (38.4%)	55 (39.0%)	32 (25.3%)	
Pre-LRT	118 (51.5%)	71 (50.3%)	40 (31.7%)	
Pre-TKI	38 (16.5%)	20 (14.1%)	16 (12.6%)	

HBV, hepatitis B virus; HCV, hepatitis C virus; BCLC, Barcelona Clinic Liver Cancer; AFP, alpha-fetoprotein; DCP, des-gamma-carboxy prothrombin; LRT, locoregional therapy; CR, complete response; PR, partial response; SD, stable disease; PD, progressive disease; TKI, tyrosine kinase inhibitor

The median follow-up of patients was 22.8 [20.18–25.41] months. The median time to progression (TTP) for patients with primary resistance, secondary resistance, and durable response was 2.83 [2.56–3.09] months, 11.93 [10.45–13.40] months, and not reached, respectively (primary resistance versus secondary resistance and durable response, HR 539.20 [145.78–1994.30], *p* < 0.001; secondary resistance versus durable response, HR 40.20 [17.59–91.89], *p* < 0.001) (Figure S1A). The median overall survival (OS) was 12.83 [10.36–15.30] months, 31.53 [28.09–34.97] months and not reached in primary resistance, secondary resistance, and durable response group, respectively (primary resistance versus secondary resistance and durable response, HR 4.96 [3.7–6.67], *p* < 0.001; secondary resistance versus durable response, HR 7.23 [3.30–15.83], *p* < 0.001) (Figure S1B).

### Characteristics of primary resistance

**Fig. 2:**
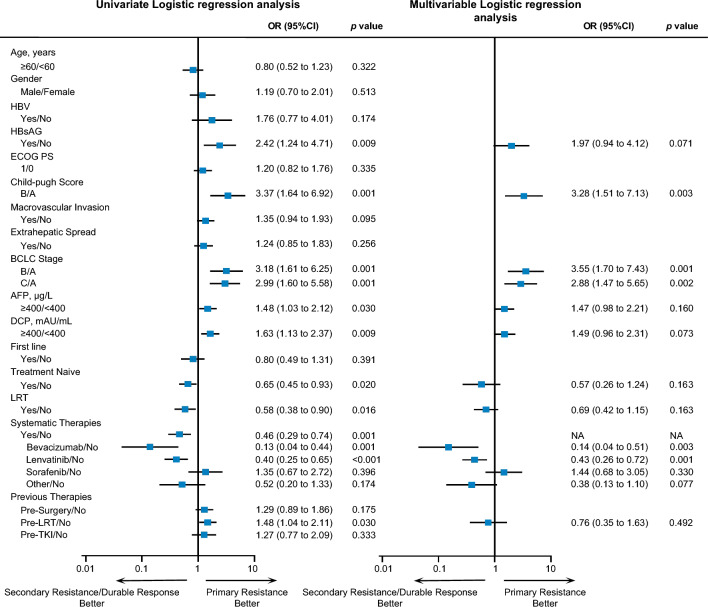
Characteristics of patients with primary resistance to ICI. Odds ratios (ORs) were calculated via univariate logistic regression analysis (left) and multivariate logistic regression analysis (right). OR, odds ratio; CI, confidence interval; NA, not applicable

The patients with primary resistance to ICI treatment exhibited significantly worse outcomes than those with secondary resistance and durable response (Figure S1). Notably, primary resistance demonstrated a stronger association with OS than secondary resistance or durable response in both time-invariant (main effect) and time-varying analyses. However, the impact of primary resistance on OS decreased over time when modeled as a time-varying covariate. In contrast, secondary resistance showed no significant correlation with OS compared to durable response in either analysis type (Table S1). Thus, we first compared the clinical characteristics between patients with primary resistance and those with secondary resistance and durable response to identify unique features of primary resistance (Fig. 2). The results showed that HBsAg-positive patients had a significantly higher risk of primary resistance to ICI therapy compared with HBsAg-negative patients (OR 2.42 [1.24–4.71], *p* = 0.009). Patients with Child–Pugh B or higher AFP/DCP levels at baseline were prone to develop primary resistance (Child–Pugh B versus A, OR 3.37 [1.64–6.92], *p* = 0.001; AFP ≥ 400 μg/L versus AFP < 400 μg/L, OR 1.48 [1.03–2.12], *p* = 0.030; DCP ≥ 400 μg/L versus DCP < 400 μg/L, OR 1.63 [1.13–2.37], *p* = 0.009). In addition, a remarkably higher risk of primary resistance was also observed in patients with BCLC stage B or C HCC (B versus A, OR 3.18 [1.61–6.25], *p* = 0.001; C versus A, OR 2.99 [1.60–5.58], *p* = 0.001). Moreover, patients treated with the combination of ICI plus locoregional therapy (LRT) or ICI plus systemic therapies (bevacizumab or TKIs) displayed a lower risk of primary resistance (LRT^+^ versus LRT^−^, OR 0.58 [0.38–0.90], *p* = 0.016; Systemic therapies^+^ versus Systemic therapies^−^, OR 0.46 [0.29–0.74], *p* = 0.001). Subgroup analysis of systemic therapies further showed that patients receiving the combination of either bevacizumab or lenvatinib plus ICI were less likely to develop primary resistance compared with those treated with ICI monotherapy (bevacizumab versus monotherapy, OR 0.13 [0.04–0.44], *p* = 0.001; lenvatinib versus monotherapy, OR 0.40 [0.25–0.65], *p* < 0.001). Meanwhile, treatment-naïve HCC was also significantly associated with a lower risk of primary resistance (OR 0.65 [0.45–0.93], *p* = 0.02). Besides, patients with previous LRT were also susceptible to primary resistance (OR 1.48 [1.04–2.11], *p* = 0.03). Moreover, the multivariate logistic regression model revealed that the Child–Pugh score, BCLC stage, and Systemic therapies were independently associated with primary resistance (Child–Pugh B versus A, OR 3.28 [1.51–7.13], *p* = 0.003; BCLC B versus A, OR 3.55 [1.70–7.43], *p* = 0.001; BCLC C versus A, OR 2.88 [1.47–5.65], *p* = 0.002; lenvatinib versus monotherapy, OR 0.43 [0.26–0.72], *p* = 0.001; bevacizumab versus monotherapy, OR 0.14 [0.04–0.51], *p* = 0.003).

### Characteristics of secondary resistance

**Fig. 3:**
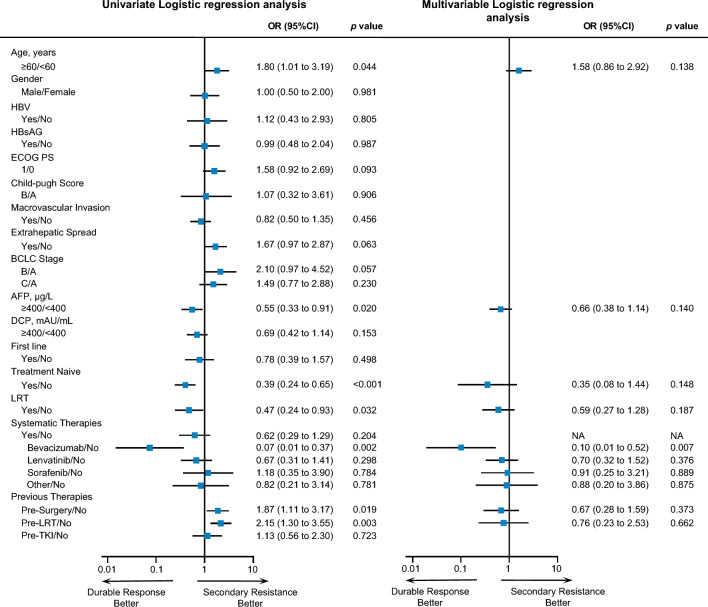
Characteristics of patients with secondary resistance to ICI. ORs were calculated via univariate logistic regression analysis (left) and multivariate logistic regression analysis (right)

We next compared the clinical characteristics between patients with secondary resistance and those with durable responses (Fig. 3). The data showed that an elder age was associated with a higher risk of secondary resistance (OR 1.80 [1.01–3.19], *p* = 0.044), while the lower risk was linked to elevated AFP levels (OR 0.55 [0.33–0.91], *p* = 0.02). A lower risk of secondary resistance was observed in patients treated with the combination of ICI plus LRT (OR 0.47 [0.24–0.93], *p* = 0.032) or ICI plus bevacizumab (OR 0.07 [0.01–0.37], *p* = 0.002). Treatment-naïve HCC was also significantly associated with a lower risk of secondary resistance (OR 0.39 [0.24–0.65], *p* < 0.001). Besides, previous surgery or previous LRT also correlated with the risk of secondary resistance (preSurgery^+^ versus preSurgery^−^, 1.87 [1.11–3.17], *p* = 0.019; preLRT^+^ versus preLRT^−^, OR 2.15 [1.30–3.55], *p* = 0.003). Moreover, the multivariate logistic regression model discovered that only the combination of ICI plus bevacizumab was independently associated with secondary resistance (bevacizumab versus monotherapy, OR 0.10 [0.01–0.52], *p* = 0.007).

### Post-progression survival

**Fig. 4:**
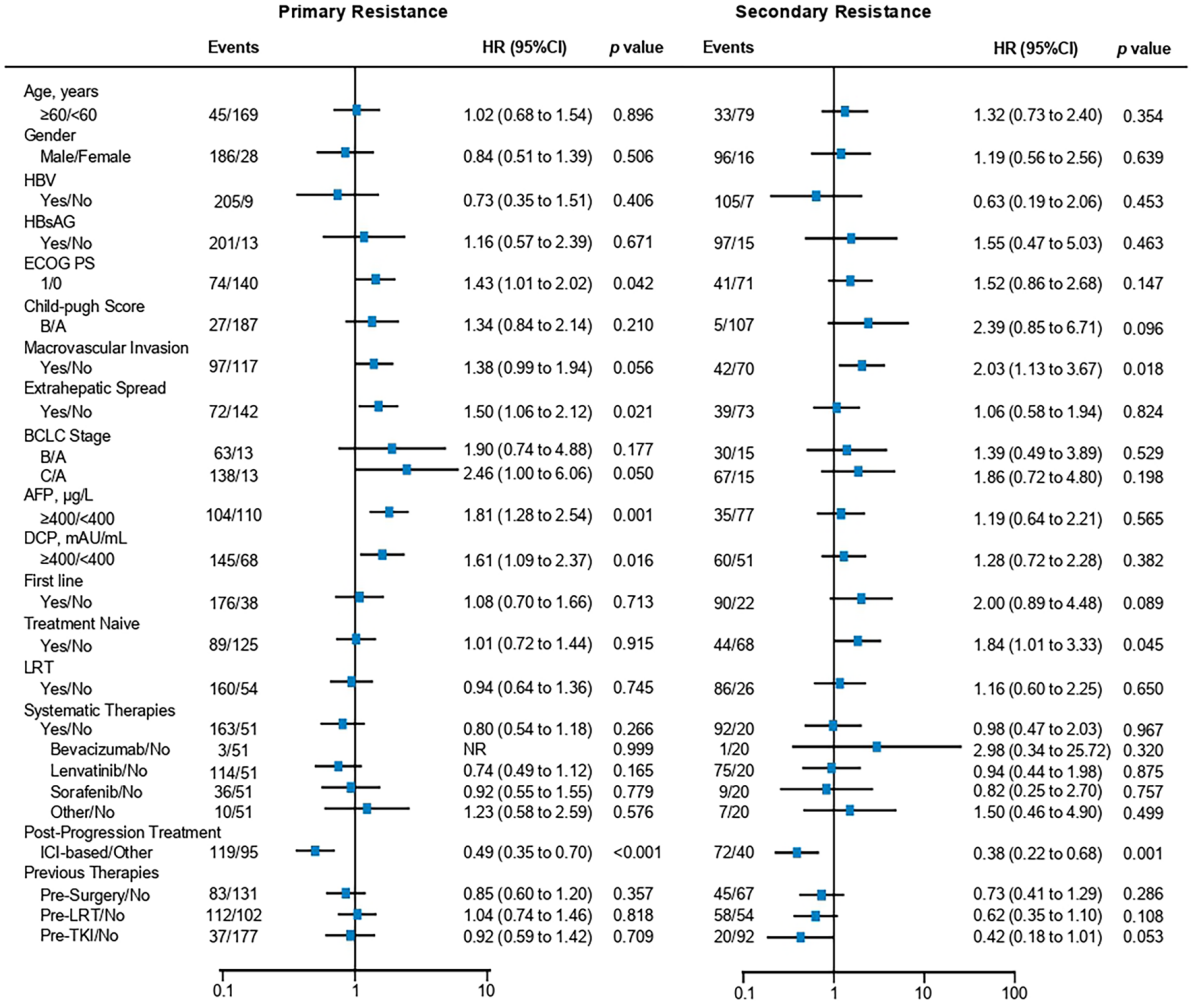
Subgroup analysis of post-progression survival (PPS) in patients with primary (left) or secondary resistance (right). The data were presented as forest plots via univariate COX regression analysis

Next, we further investigated the outcome of patients after resistance/progression. Univariate analysis in patients with primary resistance showed that patients with poor ECOG performance, extrahepatic metastasis, or higher AFP or DCP levels had a significantly poor PPS (ECOG PS 1 versus ECOG PS 0, HR 1.43 [1.01–2.02], *p* = 0.042; extrahepatic metastasis^+^ versus extrahepatic metastasis^−^, HR 1.50 [1.06–2.12], *p* = 0.021; AFP ≥ 400 μg/L versus AFP < 400 μg/L, HR 1.81 [1.28–2.54], *p* = 0.001; DCP ≥ 400 μg/L versus DCP < 400 μg/L, HR 1.61 [1.09–2.37], *p* = 0.016) (Fig. 4). Notably, post-progression therapies were also associated with PPS in patients with primary resistance (ICI-based therapy versus other therapy, HR 0.49 [0.35–0.70], *p* < 0.001) (Fig. 4). The median post-progression survival (PPS) was 9.2 [7.2–11.9] months and 19.2 [14.2–25.8] months in patients with primary and secondary resistance (primary versus secondary, HR 1.59 [1.15–2.20], *p* = 0.005), respectively (Figure S2).

**Fig. 5:**
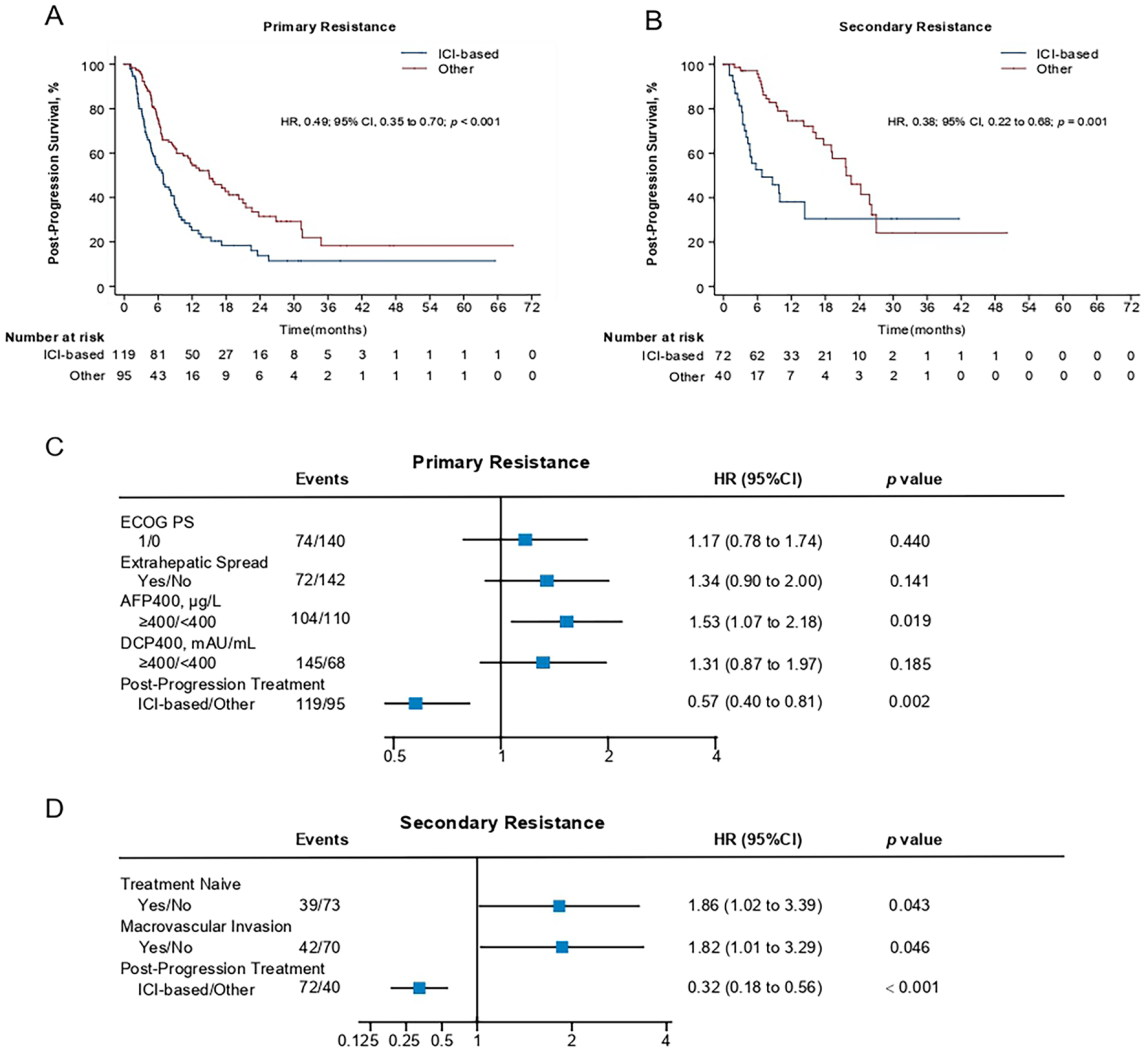
Kaplan–Meier estimates of the post-progression survival (PPS) of patients treated with ICI-based therapy or other therapies in the primary resistance group (**A**) or secondary resistance group (**B**). Characteristics of PPS in patients with primary resistance (**C**) or secondary resistance (**D**). HRs were calculated via univariate or multivariate  COX regression analysis. HR, hazard ratio; CI, confidence interval

For patients with secondary resistance, univariate analysis showed that macrovascular invasion (MacVI), or treatment-naïve HCC displayed significantly worse PPS (MacVI^+^ versus MacVI^−^, HR 2.03 [1.13–3.67], *p* = 0.018; treatment-naïve HCC versus treatment-experienced, HR 1.84 [1.01–3.33], *p* = 0.045) (Fig. 4). Moreover, post-progression therapies were also associated with PPS in patients with secondary resistance (ICI-based therapy versus other therapy, HR 0.38 [0.22–0.68], *p* = 0.001) (Fig. 4). Consistently, ICI-based post-progression therapy remarkably improved PPS in patients with primary and secondary resistance (Fig. 5A, B). The detailed ICI-based therapies after progression have been listed (Table S3, S4).

Multivariate COX analysis of PPS further revealed that AFP levels (AFP ≥ 400 μg/L versus AFP < 400 μg/L, HR 1.53 [1.07–2.18], *p* = 0.019) and post-progression therapies (ICI-based therapy versus other therapy, HR 0.57 [0.40–0.81], *p* = 0.002) were independently associated with PPS in patients with primary resistance, while post-progression therapies (ICI-based therapy versus other therapy, HR 0.32 [0.18–0.56], *p* < 0.001), treatment naïve (treatment naive versus treatment-experienced, HR 1.86 [1.02–3.39, *p* = 0.043]), and macrovascular invasion (macrovascular invasion versus no macrovascular invasion, HR 1.82 [1.01–3.29, *p* = 0.046]) were independently associated with PPS in patients with secondary resistance (Fig. 5C, D).

## Discussion

Advancements in immunotherapy have recently revolutionized the management of advanced HCC [21]. However, with the extensive use of ICI-based therapy in HCC, resistance has emerged as a key barrier in clinical practice, limiting the efficacy and durability of immunotherapy. Early identification of resistance risks before ICI treatment and tailoring the strategies to avoid or overcome resistance are of great significance. Although multiple studies have investigated the mechanism of immunotherapy resistance in HCC, they are mostly based on cellular experiments and animal models, lacking detailed clinical characteristics of patients with resistance from the real world [11, 14, 15, 22]. In this study, using a large cohort of HCC patients on immunotherapy, we comprehensively described the outcomes and characteristics of patients with resistance, finding nearly three-quarters of HCC patients developed immunotherapy resistance (46.17% primary, 28.43% secondary). Primary resistance indicated the worst prognosis, and durable response indicated the best prognosis. Child–Pugh B was an independent risk factor for primary resistance. BCLC stage A and ICI combined with bevacizumab or lenvatinib independently correlated with the lower risk of primary resistance. On the other hand, patients receiving the combination of ICI plus bevacizumab were also at a lower risk of secondary resistance.

Child–Pugh B patients with HCC, unlike their Child–Pugh A counterparts, have reduced liver reserves and systemic therapy tolerance. Altered drug metabolism and distribution may lower drug concentrations, heightening primary resistance risks [17, 23]. Persistent hepatic inflammation in Child–Pugh B patients can also activate pathways like IL-6/STAT3 and IL-17, promoting tumor cell proliferation, invasion, and drug resistance [24, 25]. A recent meta-analysis reported that the HR of survival between Child–Pugh B versus Child–Pugh A was 1.65 (*p* = 0.4502) in HCC patients treated with ICI [26]. Consistently, our findings also demonstrated that patients with liver dysfunction at baseline were at a higher risk of primary resistance, highlighting the importance of preserved liver function for immunotherapy in patients with HCC.

A meta-analysis has demonstrated that compared with non-viral-associated HCC, patients with HBV-associated HCC had a more potent immunosuppressive milieu and were more likely to benefit from immunotherapy [27]. However, our study found no link between HBV-associated HCC and lower resistance risk. Instead, HBsAg status correlated with higher primary resistance risk, implying HBV clearance and antiviral therapy might boost immunotherapy efficacy. Consistently, a recent study demonstrated that antiviral therapy considerably improves the survival of patients with HBV-related HCC [28]. On the other hand, HBV cccDNA inhibitors were observed to reduce HBV cccDNA levels, suggesting a new strategy to cure patients with chronic HBV infection [29]. Further studies with larger cohorts would be beneficial to clarify the association between HBsAg status, HBV-HCC and immunotherapy resistance.

The BCLC staging system provides a framework for treatment stratification and prognosis prediction in patients with HCC, therefore contributing to the development of novel therapeutic interventions and personalized treatment [30]. ICI-based combination therapies approved for advanced HCC still face primary resistance to immunotherapy in about 70% of patients. Our findings indicate that BCLC stages B and C were independently linked to a higher risk of primary resistance. This may stem from the elevated tumor burden and increased tumor microenvironment heterogeneity in intermediate to advanced HCCs [31]. Our finding of the association between BCLC stage and primary resistance was in line with the previous studies [32, 33], indicating that BCLC staging is also an important tool for evaluating the prognosis of HCC in the context of primary drug resistance.

Immunotherapies have evolved from monotherapy to progressively more intense dual or triplet therapy to overcome the intrinsic primary resistance of HCC. Currently, combined immunotherapy is the cornerstone of management for unresectable HCC through additive or synergistic effects [21, 34]. The EMERALD-1 phase 3 trial found that combining ICI (durvalumab) with bevacizumab and TACE significantly improved PFS over TACE alone in embolization-eligible HCC patients, indicating that ICI-based therapy plus TACE enhances clinical efficacy [35]. Similarly, our data also observed a lower risk of primary and secondary resistance in patients treated with the combination of ICI plus locoregional therapy (LRT) in comparison to ICI monotherapy. However, multivariate analysis revealed that the combination of ICI and LRT was not independently associated with resistance. On the other hand, in advanced HCC patients, atezolizumab plus bevacizumab demonstrated significant superiority over sorafenib. This synergy has established it as the first-line standard of care for advanced HCC [4, 5]. Wang et al. found that pre-existing immunity correlates with better clinical outcomes from the combination therapy of atezolizumab and bevacizumab. Key molecular correlates of combination therapy were identified, highlighting that anti-VEGF may synergize with anti-PD-L1 by targeting angiogenesis, Treg proliferation, and myeloid inflammation. The improved combinational efficacy was associated with high VEGF Receptor 2 (KDR) expression, Tregs, and bone marrow inflammatory features [36]. Consistently, compared with those treated with ICI monotherapy, patients treated with ICI in combination with bevacizumab or lenvatinib were confirmed to be less likely to develop primary resistance in our study. In addition, patients on ICI with bevacizumab, but not lenvatinib, showed lower secondary resistance, similar to the durable response seen in atezolizumab plus bevacizumab-treated patients in IMbrave 150.

According to a recent study, post-treatment AFP alterations can serve as a prognostic biomarker for patients receiving atezolizumab plus bevacizumab [37]. In addition, the high CRAFITY score based on serum AFP and C-reactive protein (CRP) indicates worse prognosis in HCC patients treated with ICIs [38, 39]. In this study, high AFP levels were associated with primary resistance to immunotherapy, though not significantly in multivariable analysis. However, high AFP level emerged as an independent prognostic factor for primary resistance in PPS analysis, offering a new biomarker for patients with primary resistance to immunotherapy. Single-cell RNA sequencing reveals that AFP-positive HCC (APHC) has an immunosuppressive microenvironment. APHC tumor cells have genes linked to antigen processing and interferon-γ response. There is also a loss of multiple T-cell subsets and a build-up of tumor-associated macrophages, making APHC more prone to drug resistance [40].

Currently, few studies have explored sequential therapies after immune resistance for patients with HCC and other tumors, and no uniform approaches for resistance management have been developed yet [41, 42]. Available guidelines recommend the use of TKIs after progression on first-line atezolizumab plus bevacizumab [43]. A phase 3 study, IMbrave251, is now evaluating atezolizumab plus lenvatinib or sorafenib versus lenvatinib or sorafenib alone in HCC progressed on atezolizumab plus bevacizumab (NCT04770896). On the other hand, several studies reported that patients with lung or renal cell carcinoma can still benefit from the subsequent anti-PD-(L)1 therapy after resistance to immunotherapy [44–46]. Qin et al. reported that substantial tumor shrinkage was found in patients with advanced HCC who continued to use camrelizumab even after disease progression, suggesting that ICIs could still be beneficial for certain patients after disease progression [47]. Furthermore, Talbot et al. explored the sequential treatment after PD of ICI-based therapy and revealed that the continuation of ICI therapy or switching to TKI therapy after PD predicted prolonged PPS [48]. Consistently, we also discovered that the continuation of ICI-based therapies remarkably improves the prognosis of HCC patients with either primary or secondary resistance, highlighting the pivotal role of immunotherapy in the sequential management of resistance. In patients with primary resistance to certain therapies, switching to another class of treatment is usually preferred. Notably, ICI-based therapy was also associated with improved PPS in patients with primary resistance. Several potential mechanisms could explain this. First, immune re-activation may play a key role. In both primary and secondary resistance, ICI-based post-progression treatments may improve PPS through mechanisms that reactivate anti-tumor immunity, such as reactivating exhausted T cells or altering the tumor microenvironment [49]. The tumor immune microenvironment may change over time, allowing ICIs to be effective again [20]. Another possible explanation was that the PD pattern in patients with primary resistance to ICIs. Recent studies revealed that HCC patients with less aggressive PD pattern after progressed to ICIs might still benefit from subsequent therapies and display improved outcomes. To further clarify the causality, prospective studies stratifying by resistance mechanisms (e.g., IFN-γ pathway loss vs. antigen presentation defects) are needed [50].

Briefly, the incidence of treatment-related adverse effect (TRAE) was 46.03% (58/126) in the durable response group, 30.57% (70/229) in the primary resistance group, and 46.81% (66/141) in the primary resistance group, respectively (Table S2). Kaplan–Meier analysis revealed that in the primary resistance group, patients with TRAEs exhibited superior OS compared to those without TRAEs (*p* = 0.001). In contrast, no significant difference in OS was observed between patients with and without TRAEs in the durable response group (*p* = 0.806) or secondary resistance group (*p* = 0.59) (Figure S3A–C).

Evidence is mounting that TRAEs from ICI-based therapies may indicate a better prognosis in various tumors, including HCC. Our study also found that TRAEs were associated with an improved prognosis in the primary resistance group, implying these patients can still benefit from ICIs. However, no such link was found in the durable response or secondary resistance groups. Michielin et al. conducted a time-dependent analysis and showed no significant association between the late-onset, long-lasting irAEs and OS in lung cancer and melanoma patients [51]. Prolonged ICI exposure in patients from durable response or secondary resistance group may raise the incidence of TRAEs, reducing survival differences between those with and without TRAEs. However, the detailed mechanism under the discrepancy merits further investigation in larger prospective cohorts.

There is no doubt that our study has limitations. Firstly, generalizability is constrained by the HBV-dominant cohort and single-center design. Patients in HBV-endemic regions may exhibit unique comorbidities and immune profiles, limiting the applicability of findings to areas with lower HBV prevalence. The single-center design may introduce selection and information biases, as institutional protocols and patient demographics may not adequately reflect population heterogeneity. To address these limitations, future research should involve multicenter studies with diverse cohorts to validate and extend these findings. Secondly, the number of patients treated with ICI combined with bevacizumab was limited due to the high economic burden of atezolizumab and the late health-insurance coverage of bevacizumab in China (December 2021). Thirdly, due to the geographic origin, patients with HCV infection and other nonviral etiologies were few, which may limit the interpretation of results, therefore the association between HCV status and primary or secondary resistance was not analyzed in the study. Unmeasured confounders are inevitably an important potential source of statistical uncertainty in clinical studies and may be associated with both exposure factors and outcomes. The degree of bias depends on the strength of the association of the confounder with exposure and outcome. Fourthly, due to the complexity and limited data of post-resistance management in clinical practice, we only classified post-progression therapies as ICI-based and others.

In conclusion, for the first time, we present the characteristics of HCC patients who were resistant to immunotherapy and elucidate the relevant factors associated with the prognosis of patients after resistance. In particular, patients who received the combination therapy of ICI plus bevacizumab were at a notably lower risk of resistance. High levels of AFP were independently associated with prognosis in patients with primary resistance to immunotherapy. ICI-based maintenance therapies may provide prominent survival benefits for HCC patients after resistance.

## Supplementary Information

Below is the link to the electronic supplementary material.

Supplementary file1 (DOCX 564 kb)

## Data Availability

The data that support the findings of this study are available from the corresponding author upon reasonable request. The data are not publicly available due to privacy or ethical restrictions.

## Abbreviations

HCC: Hepatocellular carcinoma
ICI: Immune checkpoint inhibitor
OR: Odds ratio
OS: Overall survival
PD-1: Programmed cell death-1
PPS: Post-progression survival
TTP: Time to progression
