# Supplementary material for: Characteristics and outcomes of primary and secondary resistance to immune checkpoint inhibitors in hepatocellular carcinoma
Source: Cancer Immunol Immunother. 2025 Jun 7;74(8):239. doi: 10.1007/s00262-025-04089-x (PMC12145340; doi:10.1007/s00262-025-04089-x)
Supplement: Supplementary file 1 — Supplementary file1 (DOCX 564 kb) [file 262_2025_4089_MOESM1_ESM.docx]

**Characteristics and Outcomes of Primary and Secondary Resistance to Immune Checkpoint Inhibitors in Hepatocellular Carcinoma**

Xiaowen Cui^1, #^; Minghao Ruan^2 #^; Yao Li^2, #^; Cheng Yang^3, #^; Jin Zhang^2^; Riming Jin^2^; Dong Wu^2^; Wen Sun^4, *^; Ruoyu Wang^2, *^.

^1^Department of Oncology, Eastern Hepatobiliary Surgery Hospital, the Naval Medical University, Shanghai, China.

^2^The First Department of Hepatic Surgery, Eastern Hepatobiliary Surgery Hospital, the Naval Medical University, Shanghai, China.

^3^Department of Special Treatment I and Liver Transplantation, Eastern Hepatobiliary Surgery Hospital, the Naval Medical University, Shanghai, China.

^4^National Center for Liver Cancer, the Naval Medical University, Shanghai, China.

^#^The authors have contributed equally to this work.

**Running title:**  Primary and Secondary Resistance to ICIs in HCC

***Correspondence author and address:**

Dr. Ruoyu Wang (wangruoyu1213@126.com), the First Department of Hepatic Surgery, Eastern Hepatobiliary Surgery Hospital, the Naval Medical University, 225 Changhai Road, 200438 Shanghai, China. Dr. Wen Sun (sunwen_sw@aliyun.com), National Center for Liver Cancer, the Naval Medical University, 800 Xiangyin Road, 200433 Shanghai, China.

**Table. S1 Time-adjusted Cox Regression Analysis of Overall Survival.**

| Variable |  | Overall Survival | | | |
| --- | --- | --- | --- | --- | --- |
|  |  | HR | Lower | Upper | *p* |
| Primary Resistance  versus  Secondary Resistance  + Durable Response | As Time-invariant covariate (main effect) | 25.82 | 12.12 | 54.99 | <0.001 |
|  | As Time-varying covariate | 0.90 | 0.867 | 0.94 | <0.001 |
| Secondary Resistance versus  Durable Response | As Time-invariant covariate (main effect) | 1.88 | 0.29 | 12.14 | 0.503 |
|  | As Time-varying covariate | 1.07 | 0.97 | 1.17 | 0.169 |

Time-adjusted Cox regression analysis was conducted to assess the effects of resistance on overall survival in HCC patients, comparing Primary Resistance versus Secondary Resistance+Durable Response, and Secondary Resistance versus Durable Response. In this model, *p* values were calculated from the Wald test.

**Table S2. Distribution of TRAEs across different categories.**

| TRAE | **No. (%)** | | | ***p*** |
| --- | --- | --- | --- | --- |
|  | **Primary Resistance (n=229)** | **Secondary Resistance (n=141)** | **Durable Response (n=126)** |  |
|  |  |  |  |  |
| Yes | 70 (30.5%) | 66 (46.8%) | 58 (46.0%) | 0.001 |
| No | 159 (69.5%) | 75 (53.2%) | 68 (54.0%) |  |

TRAE, treatment-related adverse effect.

**Table S3.** **lmmune Checkpoint Inhibitors Used in Patients.**

| PD-1/PD-L1 | Primary Resistance (n=229) | Secondary Resistance (n=141) | Durable Response (n=126) |
| --- | --- | --- | --- |
| Toripalimab | 88 (38.4%) | 65 (46.0%) | 37 (29.3%) |
| Sintilimab | 33 (14.4%) | 31 (21.9%) | 47 (37.3%) |
| Tislelizumab | 41 (17.9%) | 17 (12.0%) | 16 (12.6%) |
| Nivolumab | 40 (17.4%) | 8 (5.6%) | 5 (3.9%) |
| Pembrolizumab | 12 (5.2%) | 8 (5.6%) | 10 (7.9%) |
| Camrelizumab | 12 (5.2%) | 9 (6.3%) | 5 (3.9%) |
| Atezolizumab | 3 (1.3%) | 2 (1.4%) | 6 (4.7%) |
| Durvalumab | 0 | 1 (<1.0%) | 0 |

**Table S4. Detailed ICI-based therapies after progression.**

| PD-1/PD-L1 combinations | Primary Resistance (n=214) | Secondary Resistance (n=112) |
| --- | --- | --- |
| Atezolizumab (PD-L1 mAb, 1200mg)+Bevacizumab | 10 (4.6%) | 10 (8.1%) |
| Sintilimab (PD-1 mAb, 200mg)+Bevacizumab | 7 (3.2%) | 3 (2.4%) |
| Toripalimab (PD-1 mAb, 240mg)+TKI | 35 (16.3%) | 27 (22.1%) |
| Sintilimab (PD-1 mAb, 200mg)+TKI | 16 (7.4%) | 11 (9.0%) |
| Tislelizumab (PD-1 mAb, 200mg)+TKI | 13 (6.0%) | 8 (6.5%) |
| Camrelizumab (PD-1 mAb, 200mg)+TKI | 15 (7.0%) | 4 (3.2%) |
| Pembrolizumab (PD-1 mAb, 200mg)+TKI | 6 (2.8%) | 4 (3.2%) |
| Nivolumab (PD-1 mAb, 240mg)+TKI | 3 (1.4%) | 1 (<1.0%) |
| Durvalumab (PD-L1 mAb, 1500mg)+TKI | 0 | 1 (<1.0%) |
| Atezolizumab (PD-L1 mAb, 1200mg)+TKI | 1 (<1.0%) | 0 |
| Toripalimab (PD-1 mAb, 240mg) | 3 (1.4%) | 4 (3.2%) |
| Nivolumab (PD-1 mAb, 240mg) | 5 (2.3%) | 0 |
| Tislelizumab (PD-1 mAb, 200mg) | 3 (1.4%) | 1 (<1.0%) |
| Pembrolizumab (PD-1 mAb, 200mg) | 2 (<1.0%) | 1 (<1.0%) |
| Sintilimab (PD-1 mAb, 200mg) | 1 (<1.0%) | 0 |

mAb, monoclonal antibody; TKI, tyrosine kinase inhibitor.


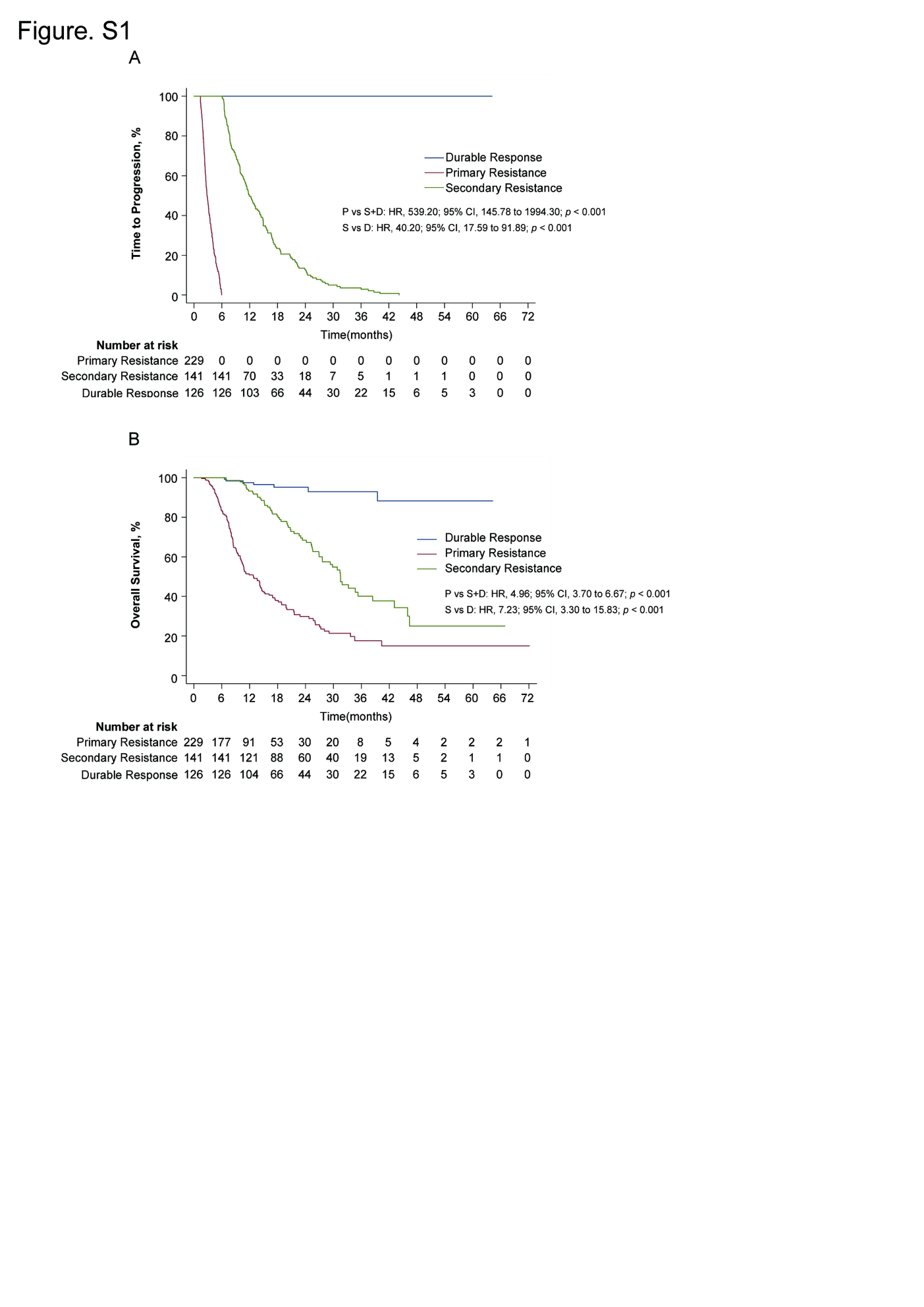


Figure S1. Kaplan-Meier estimates of time to progression (TTP) (A) and overall survival (OS) (B) in HCC patients with primary resistance, secondary resistance, or durable response to ICIs. *p* values were calculated from the log-rank test. P, primary resistance; S, secondary resistance; D, durable response.


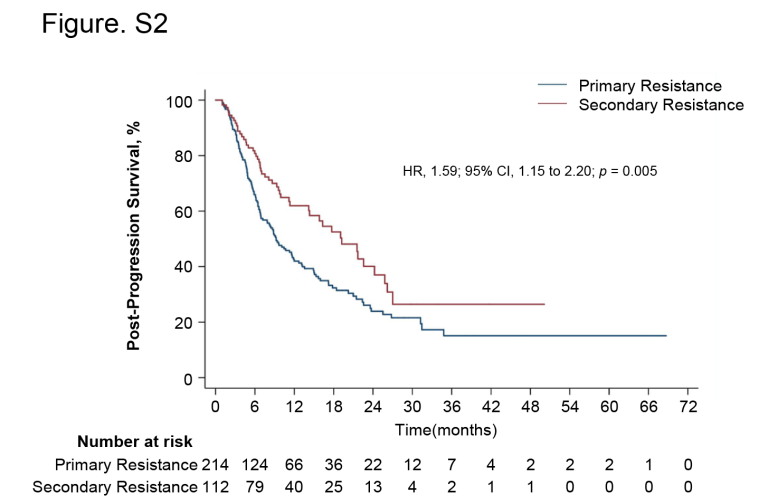


Figure S2. Kaplan-Meier estimates of the post-progression survival (PPS) in patients with primary or secondary resistance to ICIs. *p* values were calculated from the log-rank test.


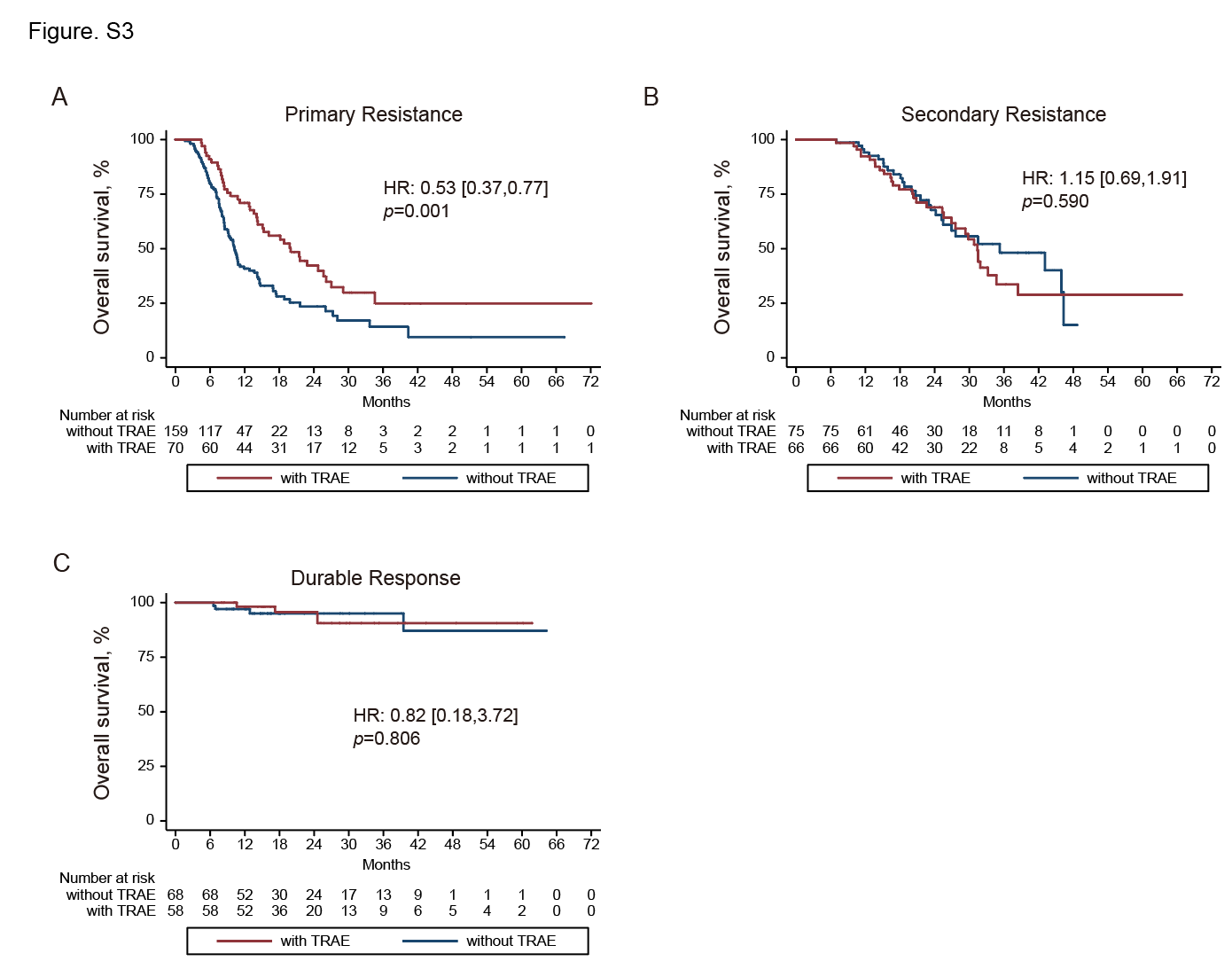


Figure S3. Kaplan-Meier estimates of the overall survival in patients with or without TRAEs. *p* values were calculated from the log-rank test.
